# Evaluating clinical and prognostic implications of Glypican-3 in hepatocellular carcinoma

**DOI:** 10.18632/oncotarget.12066

**Published:** 2016-09-16

**Authors:** Ahmed Omar Kaseb, Manal Hassan, Sahin Lacin, Reham Abdel-Wahab, Hesham M. Amin, Ahmed Shalaby, Robert A. Wolff, James Yao, Asif Rashid, Bharathi Vennapusa, Janine Feng, Toshihiko Ohtomo

**Affiliations:** ^1^ Department of Gastrointestinal Medical Oncology, The University of Texas MD Anderson Cancer Center, Houston, Texas, USA; ^2^ Division of Pathology, The University of Texas MD Anderson Cancer Center, Houston, Texas, USA; ^3^ Department of Medical Oncology, Hacettepe University, Medical Faculty, Ankara, Turkey; ^4^ Department of Clinical Oncology, Assiut University Hospital, Assiut, Egypt; ^5^ Ventana Medical Systems, Inc., Tucson, Arizona, USA; ^6^ Chugai Pharmaceutical Co., LTD., Tokyo, Japan

**Keywords:** hepatocellular carcinoma, glypican-3, immunohistochemistry

## Abstract

Hepatocellular carcinoma (HCC) is one of the most deadly cancers worldwide. In patients with HCC, histopathogical differentiation is an important indicator of prognosis; however, because determination of HCC differentiation is difficult, the recently described immunohistochemical (IHC) marker glypican3 (GPC3) might assist in HCC prognostication.The goal of our study was to investigate GPC3's IHC staining pattern and define the relationship between its expression and patients' clinicopathologic features and overall survival. We retrieved clinical parameters from 101 pathologically diagnosed HCC patients' medical records and classified these patients into 4 clinical score categories (0–3) based on increasing GPC3 staining intensity and the percentage of stained tumor cells in their resection and biopsy specimens. Histopathological samples were well, moderately, and poorly differentiated in 33, 22, and 12 patients, respectively, and the GPC3 expression rate was 63%, 86%, and 92%,respectively. The median overall survival was 49.9 months (confidence interval (CI): 35.3–64.6 months) for clinical scores 0–1 and 30.7 months (CI: 19.4–41.9 months) for clinical scores 2–3. This difference was not statistically significant (*P* = .06) but showed a strong trend. In conclusion, a greater GPC3 expression is associated with a worse HCC prognosis and may be a promising prognostic marker.

## INTRODUCTION

Hepatocellular carcinoma (HCC) is the second most common cause of cancer-related mortality worldwide, and it is the fifth most common cancer in men (554,000 cases/year, 7.5% of total cancer cases) and the ninth in women (228,000 cases/year, 3.4% of total cancer cases). Each year, 745,000 people worldwide die of the disease [1]. Cirrhosis is the greatest single risk factor for HCC, and other risk factors include chronic hepatitis B or C infection, exposure to aflatoxin, alcohol abuse, fatty liver disease, and smoking. HCC is commonly diagnosed at advanced stages, by which time it is usually incurable, unless amenable for surgical intervention [2, 3]. Patient prognosis is mainly dependent upon the size and number of tumor nodules, the presence or absence of portal venous invasion, and histopathological differentiation. However, in some cases, the distinction of tumor differentiation is challenging based on histologic grounds alone. Therefore, immunohistochemical (IHC) markers have been studied for prognostication in HCC.

Because HCC has a distinctive immunohistochemical (IHC) pattern, some IHC markers, such as Glypican-3 (GPC3), hepatocyte paraffin 1 (HepPar1), polyclonal CEA (pCEA), MOC-31, CD10, and α-fetoprotein (AFP), have been used to differentiate HCC from other tumors and to evaluate prognosis [4–18]. Glypican 3 and HepPar1, for example, are useful in distinguishing high-grade dysplastic nodules from early HCC [19–24]. Well- and moderately-differentiated HCCs stain positive for HepPar1, cytoplasmic thyroid transcription factor-1, glutamine synthetase, GPC3, and cytokeratin 8/18 [21, 22, 25–27]. However, the specificity and sensitivity of these markers is limited [28], and poorly differentiated HCCs may lose immunoreactivity to some IHC markers, including HepPar1, canalicular pCEA, canalicularCD10, and AFP. There is therefore a need for more specific and sensitive immunoreactive hepatocytic markers that can recognize poorly differentiated HCC.

GPC3 shows promise as a prognostic immunohistopathological marker for HCC. GPC3 is a member of the glypican family, which has six members (GPC1-6) and is characterized by heparan sulfate proteoglycans bound to the cell surface by a glycosylphosphatidylinositol anchor [29–34] and which influences cell growth, differentiation, and migration [35–38]. Studies have shown that GPC3's expression rate in non-liver tumors, including mesotheliomas, ovarian tumors, breast tumors, and cholangiocarcinomas, is down-regulated, whereas it is high in HCC [34, 39–44]. Methods employed in assessing GPC3 expressed included immunohistochemistry (IHC), *in-situ* hybridization (ISH), real-time polymerase chain reaction (RT-PCR) and northern blot. Yamauchi N et al. assessed GPC3 expression in cell membrane and cytoplasm using IHC and categorized cases into either focally positive (+) showing 10–50% or diffusely positive (++) showing > 50% expression [43]. However, the relationship between GPC3 overexpression and prognosis has not yet been clarified in HCC.

A recent study showed that GPC3 is more sensitive than HepPar1 in detecting HCC [45]. It is especially useful in distinguishing hepatic adenomas or high-grate dysplastic nodules from well-differentiated HCC, in non-cirrhotic patients with advanced HCC [20, 43, 46–48].

Firstly, germline GPC3 mutations have been found in patients with Simpson-Golabi-Behmel syndrome, [31, 49–53]. This syndrome as an X-linked disorder that is characterized by prenatal and postnatal cellular proliferation and that includes visceral and skeletal abnormalities. Notably, some studies have reported GPC3 as a cell proliferation inhibitor and apoptosis inducer, therefore it may play a role in the prognosis of hepatocellular carcinoma [19–24, 54].

GPC3 is an oncofetal protein that is expressed in the placenta and fetal liver, but not in normal hepatic parenchyma or nonmalignant liver tissue, and it is only occasionally and weakly expressed in preneoplastic lesions. However, many studies have shown significantly increased GPC3 expression in HCC [5, 19, 20]. The goal of the current study was to determine GPC3's staining pattern in HCC and to define the diagnostic utility of GPC3 in distinguishing early from advanced HCC. We also investigated the potential prognostic value of GPC3 by analyzing the survival rates of patients with low versus high GPC3 expression in HCC tumors and determining whether GPC3 expression was associated with the patients clinicopathologic parameters.

## RESULTS

**Figure 1:**
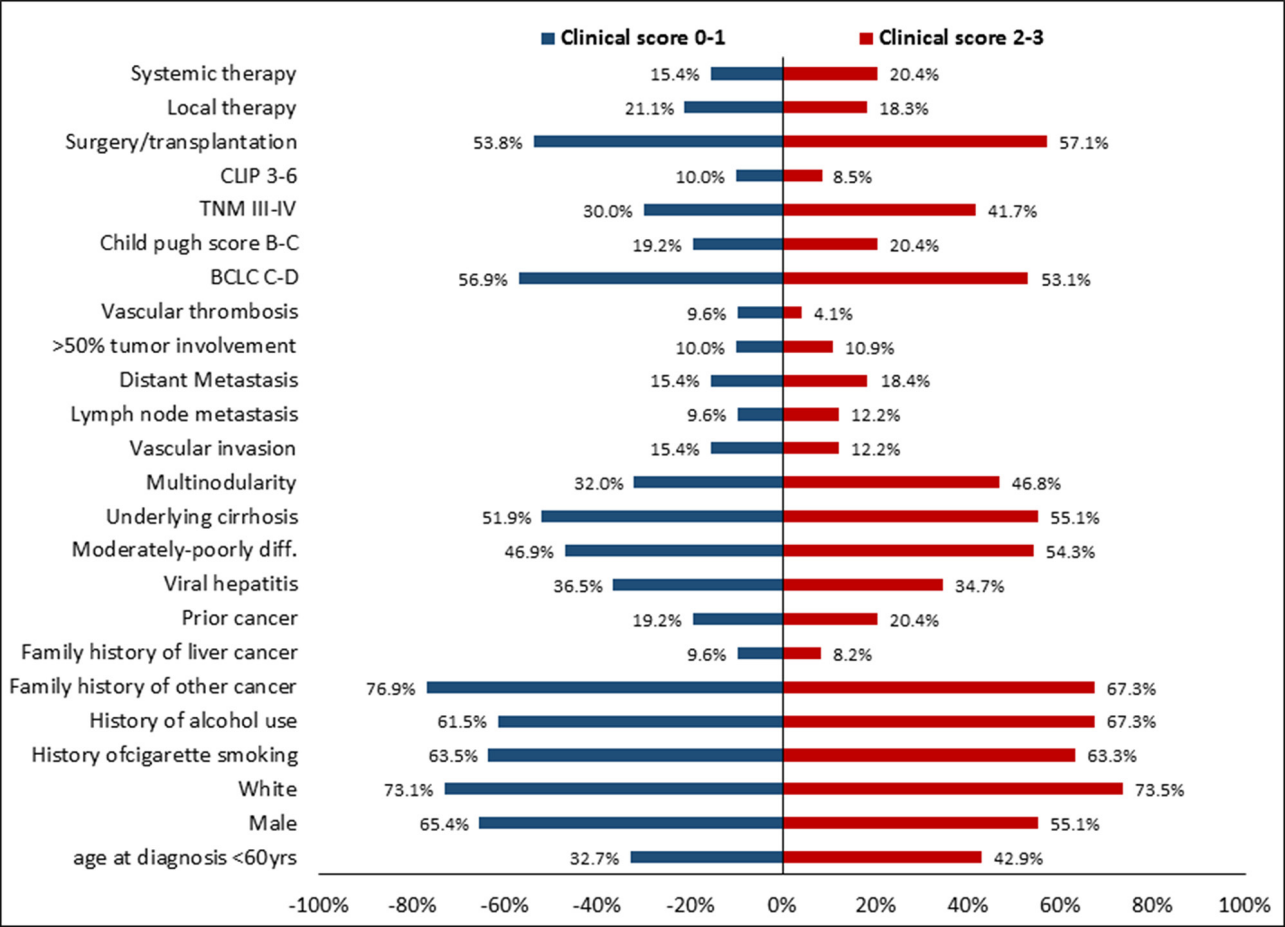
Comparison of risk factors, epidemiological parameters, demographic characteristics, and clinicopathological parameters between patients with a clinical score of 0–1 and those with a clinical score of 2–3

The detailed baseline demographic characteristics of the 101 HCC patients in our study are summarized in Table 1. The majority of patients (62.4%) were older than 60 years, with a mean age and standard deviation of 63.2 ± 11.8 years and a male-to-female ratio of 1.5:1. Risk factors for HCC were hepatitis (35.6%), alcohol consumption (64.4%). Twenty-two patients (21.8%) had extrahepatic disease (either lymph node involvement or distant metastasis). At the time of initial diagnosis, 62.4% of patients had an Eastern Cooperative Oncology Group performance status of zero, and the majority of patients had early-stage HCC according to different prognostic scoring and staging systems. Comparison between low clinical score 0–1 (*N* = 52) and high clinical score 2–3 (*N* = 49) GPC3 expression showed that there was no statistically significant difference between the two levels based on demographic characteristics, epidemiological parameters, HCC risk factors, clinicopathological characteristics, and baseline treatment modalities (Figure 1).

**Table 1:** Demographic characteristics, risk factors, and clinicopathological characteristics of 101 HCC patients

Variables	HCC patients (*N* = 101)		
	No. of patients	%	95% CI
**Age at diagnosis (years)**			
**Mean (± SD)**	63.2 ± 11.8		
** ≤ 60**	38	37.6	**0.28–0.48**
** > 60**	63	62.4	**0.52–0.72**
**Sex**			
** Male**	61	60.4	**0.5–0.7**
** Female**	40	39.6	**0.3–0.5**
**Race**			
** White**	74	73.3	**0.64–0.82**
** Non-white**	27	26.7	**0.18–0.36**
**Hepatitis status**			
** HCV only**	17	16.8	**0.1–0.26**
** HBV only**	13	12.9	**0.07–0.21**
** HCV and HBV**	6	5.9	**0.02–0.12**
** None**	65	64.4	**0.54–0.74**
**Family history of liver cancer**	9	8.9	**0.04–016**
**Family history of other cancer**	73	72.3	**0.62–0.81**
**History of other cancers**	20	19.8	**0.13–0.29**
**History of cigarette smoking**	64	63.4	**0.53–0.73**
**History of alcohol consumption**	65	64.4	**0.54–0.74**
**Presence of cirrhosis**	54	53.5	**0.43–0.63**
**Performance status (ECOG)**			
** 0**	63	62.4	**0.52–0.72**
** 1–2**	34	33.7	**0.25–0.44**
** 3–4**	4	4.0	**0.01–0.1**
**AFP level ≥ 400 ng/dl**	26	25.7	**0.18–0.35**
**Presence of vascular invasion**	14	13.9	**0.78–0.22**
> 50% tumor involvement*	10	9.9	**0.05–0.17**
**Distant metastasis**	17	16.8	**0.1–0.26**
**Lymph node metastasis**	11	10.9	**0.06–0.19**
Multi-nodularity*	38	37.6	**0.28–0.48**
Tumor differentiation*			
**Well-differentiated**	33	32.7	**0.24–0.43**
**Moderately differentiated**	22	21.8	**0.14–0.31**
**Poorly differentiated**	12	11.9	**0.06–0.2**
**Child-Pugh class**			
** A**	81	80.2	**0.71–0.87**
** B-C**	20	19.8	**0.13–0.29**
CLIP staging*			
** Stage 0–2**	88	87.1	**0.79–0.93**
** Stage 3–6**	9	8.9	**0.04–0.16**
BCLC staging*			
** Stage 0-B**	45	44.6	**0.35–0.55**
** Stage C-D**	55	54.5	**0.44–0.64**
TNM staging*			
** Stage I-II**	63	64.3	**0.52–0.72**
** Stage IIIA-IIIB**	35	35.7	**0.25–0.45**
Okuda staging*			
** Stage I**	68	67.3	**0.57–0.76**
** Stage II-III**	29	28.7	**0.2–0.39**
**Baseline treatment**			
** Surgery or transplant**	56	55.4	**0.45–0.65**
** Local therapy**	20	19.8	**0.13–0.29**
** Systemic therapy**	18	17.8	**0.11–0.27**
** Best supportive care**	7	6.9	**0.03–0.14**

Abbreviations: AFP, alpha fetoprotein; BCLC, Barcelona Clinic Liver Cancer; CLIP, Cancer of the Liver Italian Program; CI, confidence interval; ECOG, Eastern Cooperative Oncology Group; HBV, hepatitis B virus; HCV, hepatitis C virus; SD, standard deviation; TNM, tumor-node-metastasis.

* Some data are missing: > 50% tumor involvement; *N* = 5 due to unavailable radiology report, Multi-nodularity; *N* = 4 due to previous surgery, Tumor differentiation; *N* = 34 diagnosed as HCC but the grade of differentiation was not detected, CLIP; *N* = 4 due to unavailable information about tumor morphology, BCLC; *N* =1 due to unavailable information about tumor nodularity, TNM stage; *N* = 3 due to unavailable information about tumor nodularity and tumor size, OKUDA; *N* = 4 due to unavailable information about % of liver occupied by the tumor.

The Cox proportional hazard models showed that the GPC3 clinical score tended to be a significant independent risk factor for HCC OS. Compared to patients with a GPC3 clinical score of 0–1, the adjusted HR for patients with a GPC3 clinical score of 2–3 was about 1.5 times higher (adjusted hazard ratio 1.57; 95% CI, 1.007–2.47; *P* = .047, Table 2).

**Table 2:** Adjusted hazard ratios and 95% confidence intervals for glypican-3 (GPC3) clinical score and other demographic and clinicopathological characteristics and risk factors

Parameters		Adjusted HR	95%CI	*P* value
GPC3 clinical score*	0–1	1 (Reference)		
	2–3	1.57	1.007–2.47	.047
Age	≤ 60	1 (Reference)		
	> 60	1.01	0.99–1.03	.32
Sex	Female	1 (Reference)		
	Male	1.07	0.69–1.67	.76
Race	Non-white	1 (Reference)		
	White	0.9	0.54–1.62	.81
Vascular invasion	No	1 (Reference)		
	Yes	1.18	0.59–2.37	.64
Lymph node metastasis	No	1 (Reference)		
	Yes	1.44	0.61–3.44	.4
Distant metastasis	No	1 (Reference)		
	Yes	1.88	0.84–4.2	.12
% of liver occupied by tumor	≤ 50%	1 (Reference)		
	> 50%	0.8	0.39–1.65	.55
BCLC	0	1 (Reference)		
	A-B	0.59	0.3–1.16	.12
	C-D	0.74	0.42–1.28	.28
Treatment	Non-surgical	1 (Reference)		
	Surgery	0.5	0.31–0.79	.003

Abbreviations: BCLC, Barcelona Clinic Liver Cancer; CI, confidence interval; HR, hazard ratio.

* Adjusted hazard ratio of GPC3 clinical score after controlling for confounding factors such as age, sex, race, vascular invasion, lymph node involvement, distant metastasis, % of liver occupied by tumor, BCLC staging system, and HCC treatment modalities.

**Figure 2:**
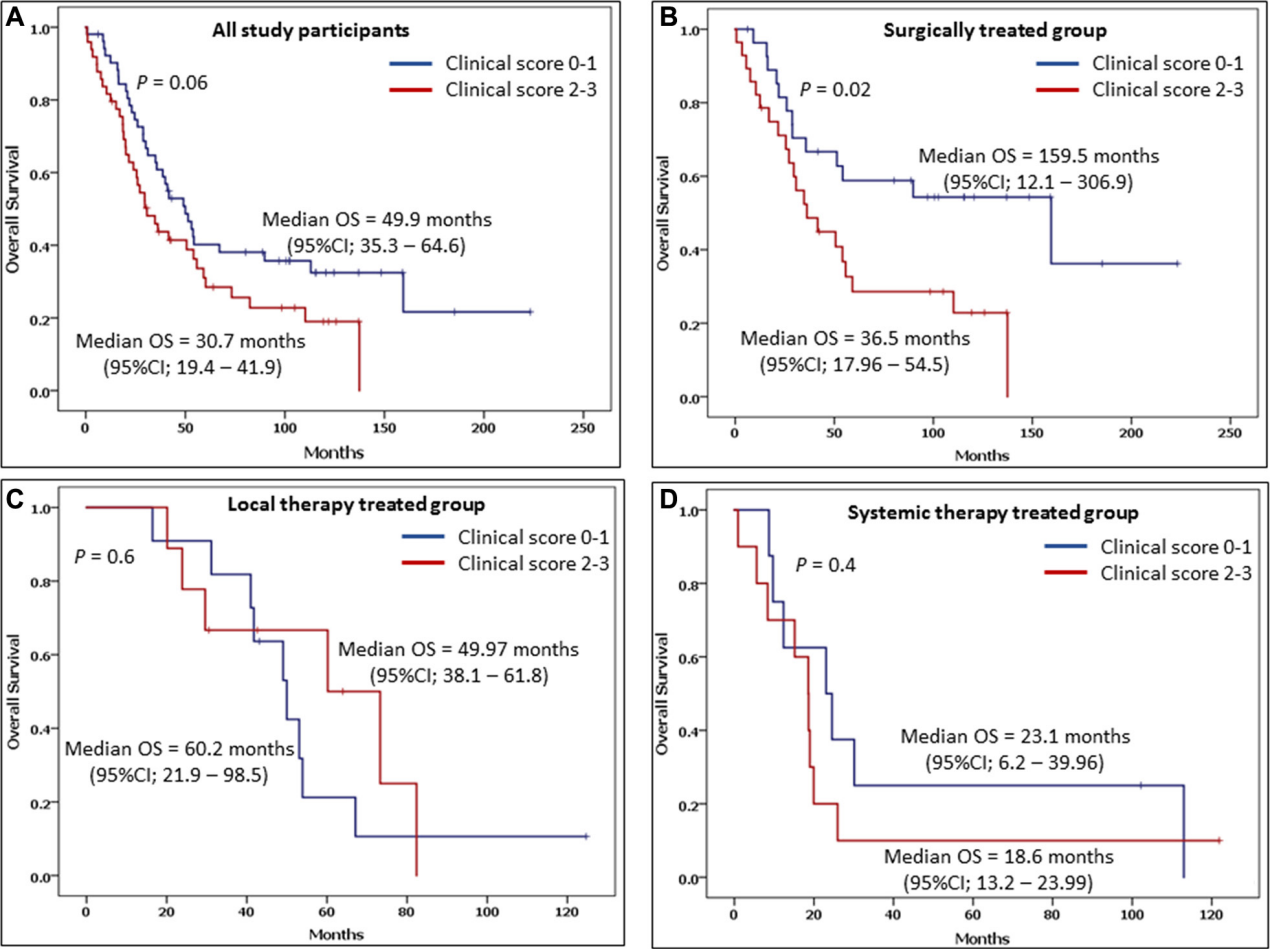
Overall survival (OS) and the 95% confidence interval (CI) in patients with clinical scores of 0–1 and 2–3 include A) among all study participants, B) among surgically treated patients, C) among local therapy treated patients, and D) among systemic therapy treated patients

Futhermore, when the OS of patients who had a clinical score of 0–1 (49.9 months [95% CI: 35.3–64.6 months]) was compared with that of those with a clinical score of 2–3 (30.7 months [95% CI: 19.4–41.9 months]), the difference was not statistically significant (*P* = 0.06)). However, comparing between varaiotion of the medican OS among patients with low and high clinical score of GPC3 based on the baseline treatment approaches showed that patients with clinical score 0–1 and treated with surgery have a significantly longer OS compared to patients with higher clinical score 2–3 (*P* = .02) (Figure 2)

**Figure 3:**
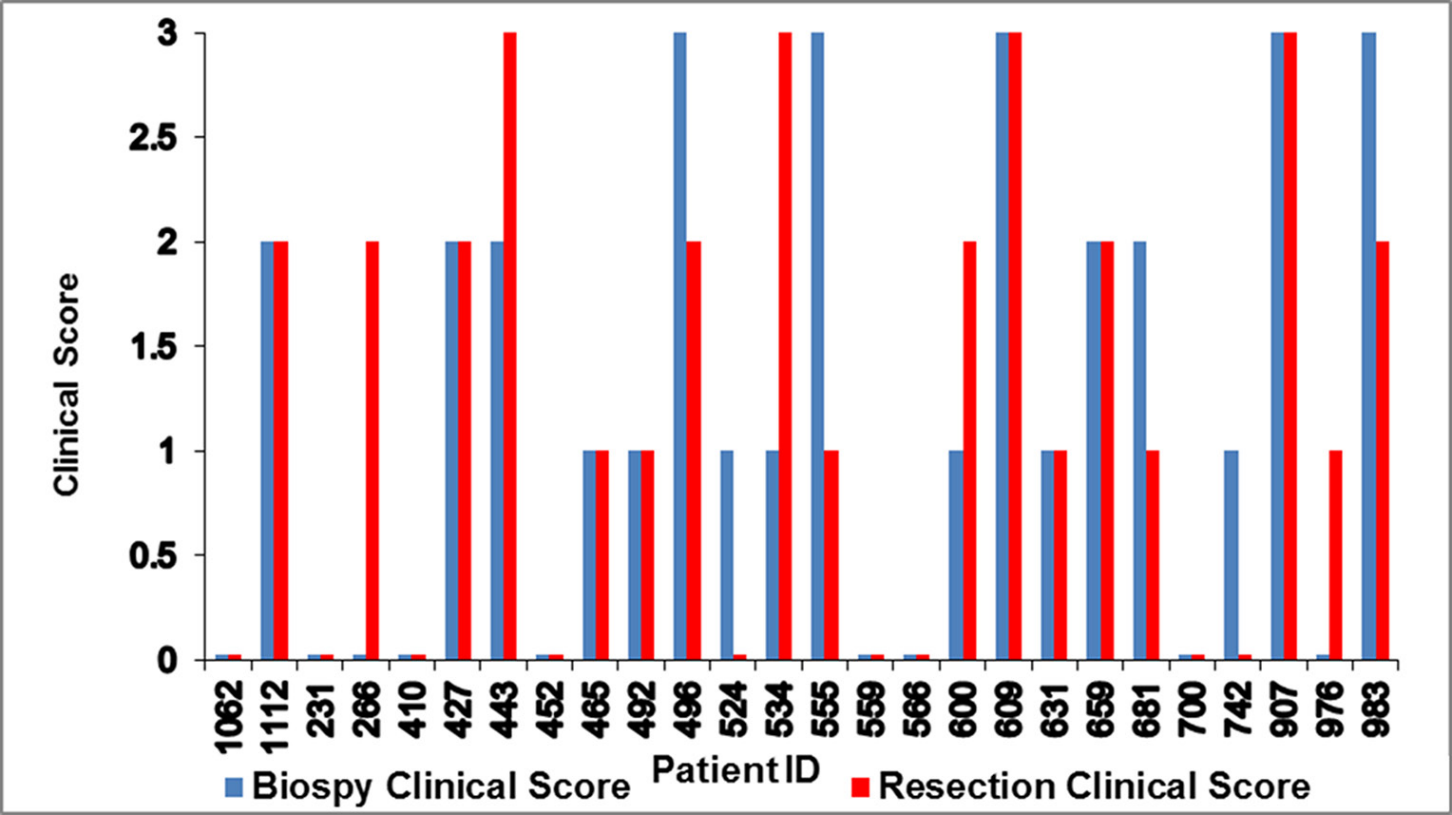
Variations in paired biopsy and resection clinical scores for 26 patients

Among the 26 patients with evaluable paired biopsy and resection samples, 15 had the same clinical score for both, even though most patients had a time gap of more than two months between the the acquision of the two samples. Six patients had a higher clinical score for the biopsy than the resection, and five patients had a higher clinical score for the resection than the biopsy (Figure 3). The concordance of clinical scores 0 vs. 1 vs. 2 vs. 3 between biopsies and resections was 57.7% (95% CI: 39.0–74.5). The concordance of clinical scores 0–1 vs. 2–3 between biopsies and resections was 80.8% (95% CI: 62.1–91.5).

## DISCUSSION

Our current study indicates that higher GPC3 expression level in HCC is a risk factor for shorter OS. GPC3 expression may also be associated with histopathologic differentiation grade and advanced clinicopathologic features, but these findings were not significant. Consistent with our findings, Yamauchi et al. found that levels of GPC3 expression in poorly differentiated tumor cells were higher than those in moderately and well differentiated tumor cells. GPC3 was expressed in 78% (14 of 18) of well-differentiated tumors, 83% (24 of 29) of moderately-differentiated tumors, and 100% (9 of 9) of poorly differentiated tumors, while the size of the HCC was not related to the level of GPC3 expression. In the same study, GPC3 expression was negative in sarcomatoid HCC, carcinoid tumors, and cholangiocarcinomas [43]. The reported sensitivity of GPC3 for HCC in the literature ranges from 75% to 100%, and in large-scale trials it ranges from 75% to 85% [26, 43, 47, 55–57].

Our study showed a large difference in OS between patients with a GPC3 clinical score of 0–1 and patients with GPC3 clinical score of 2–3. However, it wasn't statistically significant there is strong trend toward better OS. The median OS was 49.9 months (95% CI: 35.3–64.6 months) for GPC3 clinical score 0–1 and 30.7 months (95% CI: 19.4–41.9 months) for GPC3 clinical score 2–3 (*P* = 0.06) (Figure 3). Furthermore, we found a correlation between GPC3 expression rate and vascular invasion, >50% tumor involvement in the liver, lymph nodes involvement, alcohol use, cigarette smoking, race, gender, age, or stage (CLIP, TNM, BCLC and CTP). However, due to small sample size in our single institution study, this limitation may account for the lack of statistical significance in some of the correlation analyzed.

Additionally, we compared the expression of GPC3 in 26 patients who had both biopsy and resection, the majority of samples were concordant even though most of them collected after a time gap of over 2 months between the procedures. Based on this result, our study indicated that biopsies may provide reliable information about GPC3 expression. However, due to the limitations of our study, including a small number of samples, a time gap between biopsy and resection, and the effects of therapy on tissue nature, this result needs to be confirmed by future well-designed trials.

GPC3 expression and staining is diagnostically important for fibrolamellar HCC, which differs clinically, histologically, molecularly, and prognostically from conventional HCC and which typically occurs in young patients without cirrhosis. Elevation of AFP is uncommon and immunohistochemistry for AFP is generally negative in fibrolamellar HCC [58–62]. In contrast, a few trials have shown that the disease expresses and stains with GPC3, although, unlike in conventional HCC and especially poorly differentiated HCC the expression rate is not significant [5, 45].

Some studies have indicated that serum GPC3, in combination with AFP, improves diagnostic accuracy and sensitivity for early HCC [63–65]. AFP is the most commonly used serum marker for the diagnosis and detection of HCC. At a cutoff value of 20 ng/mL of serum, AFP shows a 60%–80% sensitivity in detecting tumors [66–69] that decreases to about 40% for the detection of tumors that are smaller than 3 cm [68]. Interestingly, GPC3 level is more frequently elevated than AFP level (88% versus 55%) in patients with liver cancer, and especially in those with HCC tumors < 3 cm (77% versus 43%) [70]. Thus, after independent validation, GPC3 immunoassays may be useful in diagnosing HCC, as GPC3 has been shown to have serological sensitivity and specificity of 53% and 95%, respectively [64].

Our study results suggest that the GPC3 expression rate could be a promising prognostic marker for HCC. However, large-scale, independent validation studies are warranted to confirm and further define the prognostic role and implications of GPC3 in this disease.

## MATERIALS AND METHODS

### Study design and population

Our current investigation is part of an ongoing, hospital-based, case-control study that was approved by The University of Texas MD Anderson Cancer Center Institutional Review Board. Written informed consent was obtained from each study participant.

We searched our patients database and identified 101 patients with histologically confirmed HCC treated from March 1996 to September 2012. Seven patients hadbiopsy specimens only, 31 patients had resection specimens only, and 26 patients had both resection and biopsy specimens. The following clinical variables were recorded at the time of diagnosis and retrieved from the patients' medical records: patient demographics, HCC risk factors (including cirrhosis), tumor characteristics (such as histologic differentiation, vascular invasion, extrahepatic metastasis, size and number of tumor nodules, and the percentage of the liver occupied by tumor), HCC treatment regimens and modalities, and survival. We also collected information about disease stage using various HCC staging systems, including Barcelona Clinic Liver Cancer (BCLC), Cancer of the Liver Italian Program (CLIP); tumor-node-metastasis (TNM), and OKUDA.

We retrieved the patients' paraffin-embedded tissue specimens Unstained sections, and IHC was performed at Ventana Medical Systems, Inc. (VMSI, Tucson, AZ). IHC procedures for GPC3, including antigen recovery, antibody incubation, and antibody detection, were done in the college of American pathologist (CAP) and Cliical Laboratory Improvement Act (CLIA) accredited certified laboratory. Tissue sections were stained using anti-Glypican 3 mouse monoclonal primary antibody (clone GC33, Ventana Medical Systems, Inc. Tucson, AZ) on BenchMark ULTRA to detect membrane and cytoplasmic expression. Heat-induced epitope retrieval was used followed by incubation of the primary antibody for 32 min at 36°C. Immunodetection was accomplished with ultraView Universal DAB Detection Kit. Appropriate positive and negative controls were included for each stain.

**Figure 4:**
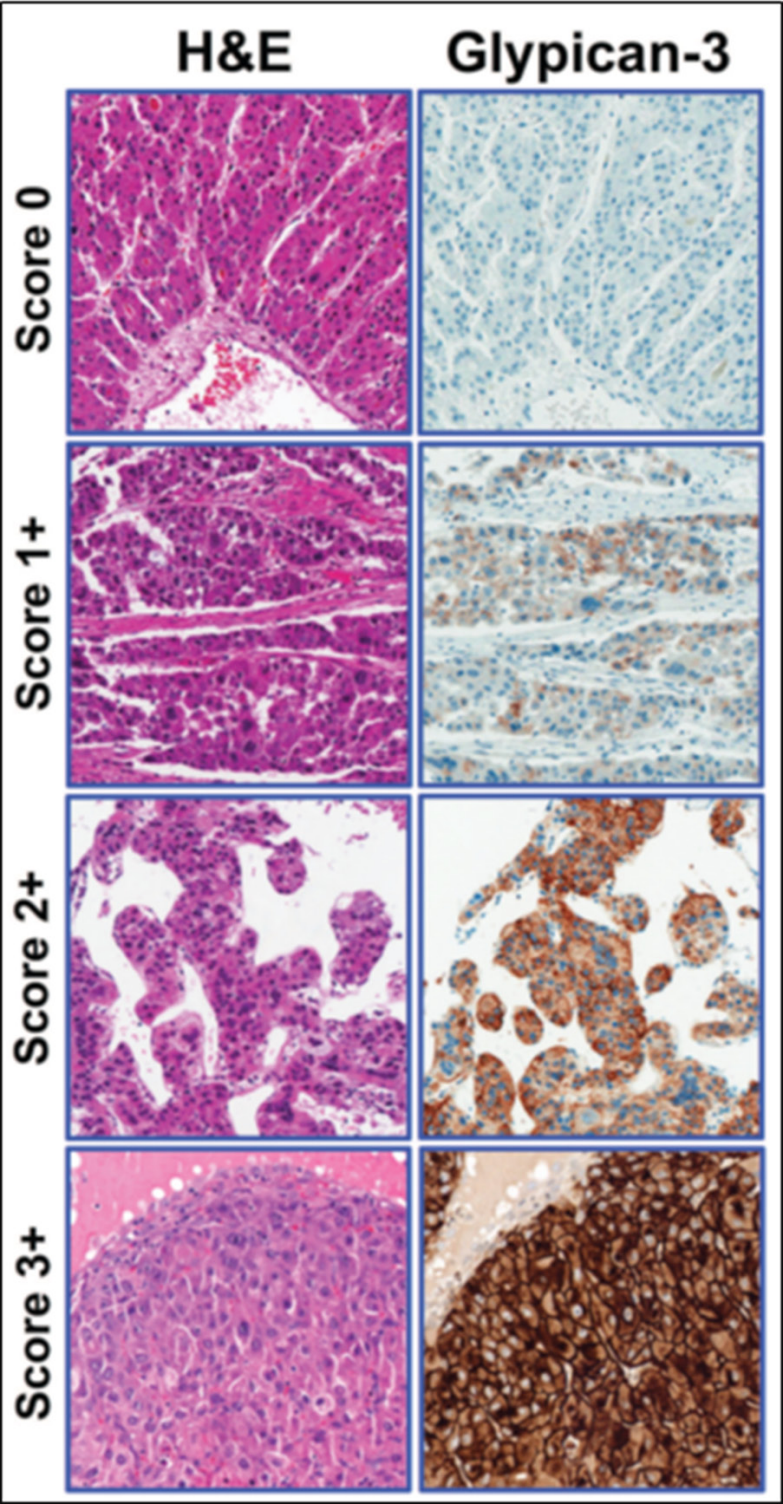
Hepatocellular carcinoma tissue samples showing the correlation between staining and clinical score categories Left panel: representative examples of hematoxylin and eosin (H&E)-stained HCC tumors. Right panel: representative examples of Glypican-3-stained HCC tumors. The immunohistochemical scores range from 0 (negative) to 3+.

Based on GPC3 staining intensity and the percentage of stained tumor cells, patients were classified into one of four clinical score categories (Figure 4) according to the criteria described in Table 3. During scoring, pathologists were blinded to specimen and clinical details.

**Table 3:** Glypican-3 (GPC3) clinical score categories

Score	Description
0	Absent membranous staining Cytoplasmic staining of any intensity in < 10% of tumor cells
1	Membranous staining of any intensity in < 10% of tumor cells, and/or Cytoplasmic staining of any intensity in > 10% of tumor cells (note that strong cytoplasmic staining, if present, must be in < 50% of tumor cells)
2	Weak to moderate membrane staining in ≥ 10% of tumor cells (note that strong membrane staining, if present, must be in < 10% of tumor cells), and/or Cytoplasmic staining of any intensity in > 10% of tumor cells (note that strong cytoplasmic staining, if present, must be in < 50% of tumor cells)
3	Strong membrane staining in > 10% of tumor cells with or without cytoplasmic staining, or Strong cytoplasmic staining in ≥ 50% of tumor cells

### Statistical methods

Stata software (Stata Corp, College Station, TX) was used for statistical analysis. Univariate analysis was done using the X^2^ or Fisher exact test for categorical variables, and the Kruskal-Wallis test was used for continuous variables for all 101 patients and for comparison between low GPC3 expression (clinical score 0–1) and high GPC3 expression (clinical score 2–3). To identify independent prognostic factors for overall survival (OS), adjusted hazard ratios (HRs) and 95% confidence intervals (CIs) were calculated using Cox proportional hazard models after adjusting for confounding factors such as age, sex, race, vascular invasion, lymph node involvement, distant metastasis, volume percentage of liver occupied by tumor, BCLC staging system, and HCC treatment modalities. The clinical score HR was adjusted for age, sex, race, vascular invasion, distant metastasis, lymph node involvement, the percentage of the liver occupied by the tumor(s), the Barcelona Clinic Liver Cancer (BCLC) staging system, and HCC treatment modalities. Survival curves were generated by the Kaplan-Meier method, and the statistical significance of differences was determined according to the log-rank test. A *P value* of .05 was considered statistically significant.
